# Supplementary material for: Telomere-to-telomere gapless chromosomes of banana using nanopore sequencing
Source: Commun Biol. 2021 Sep 7;4:1047. doi: 10.1038/s42003-021-02559-3 (PMC8423783; doi:10.1038/s42003-021-02559-3)
Supplement: Supplementary file 2 — Supplementay Information [file 42003_2021_2559_MOESM2_ESM.pdf]

Supplementary Figure 1: **KAT plot of *Musa acuminata* V4 assembly.** K-mer multiplicity in the assembly is represented by colors (black:0, red:1, purple:2, green: 3, blue: 4, orange:5).

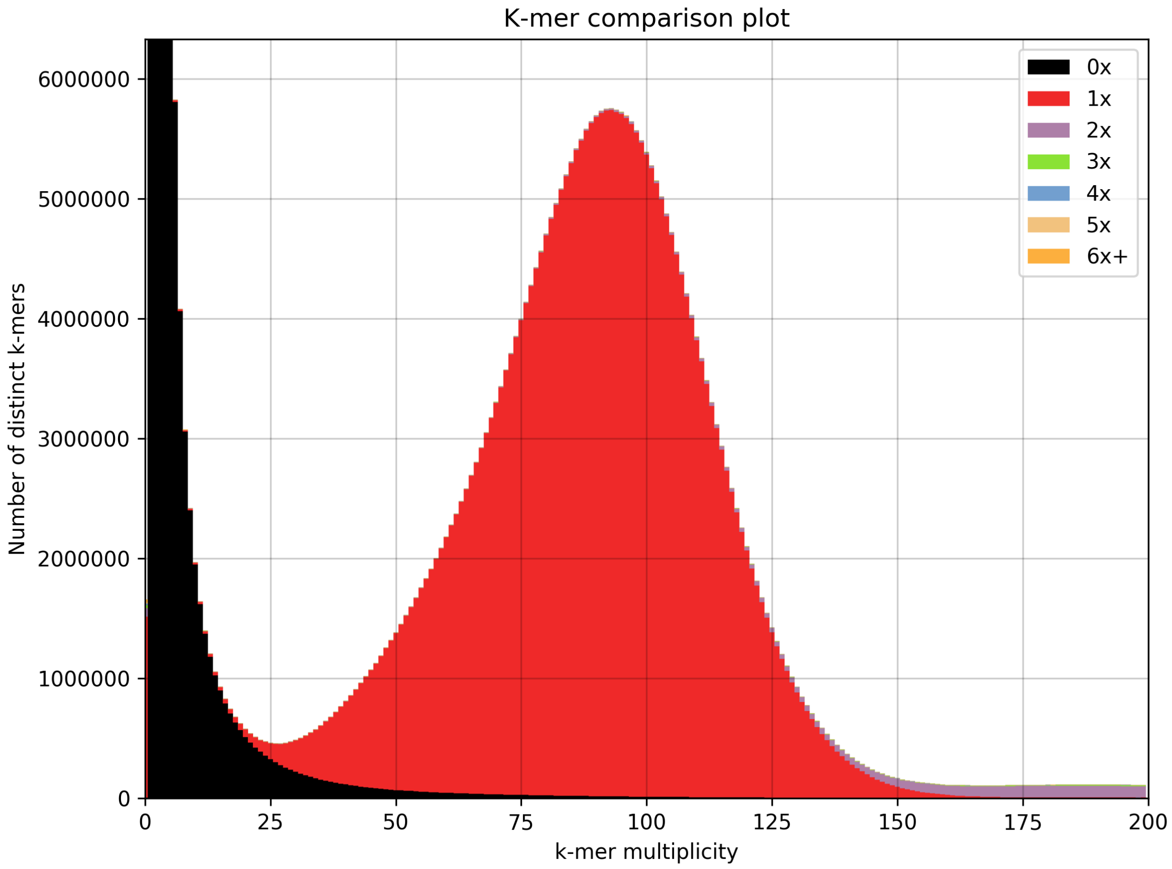

Supplementary Figure 2: Dot plot of each V4 chromosome against its relative V2 chromosome. The x axis represents the chromosome in V2. The y axis represents the chromosome in V4. The number of the chromosome is printed in brackets.

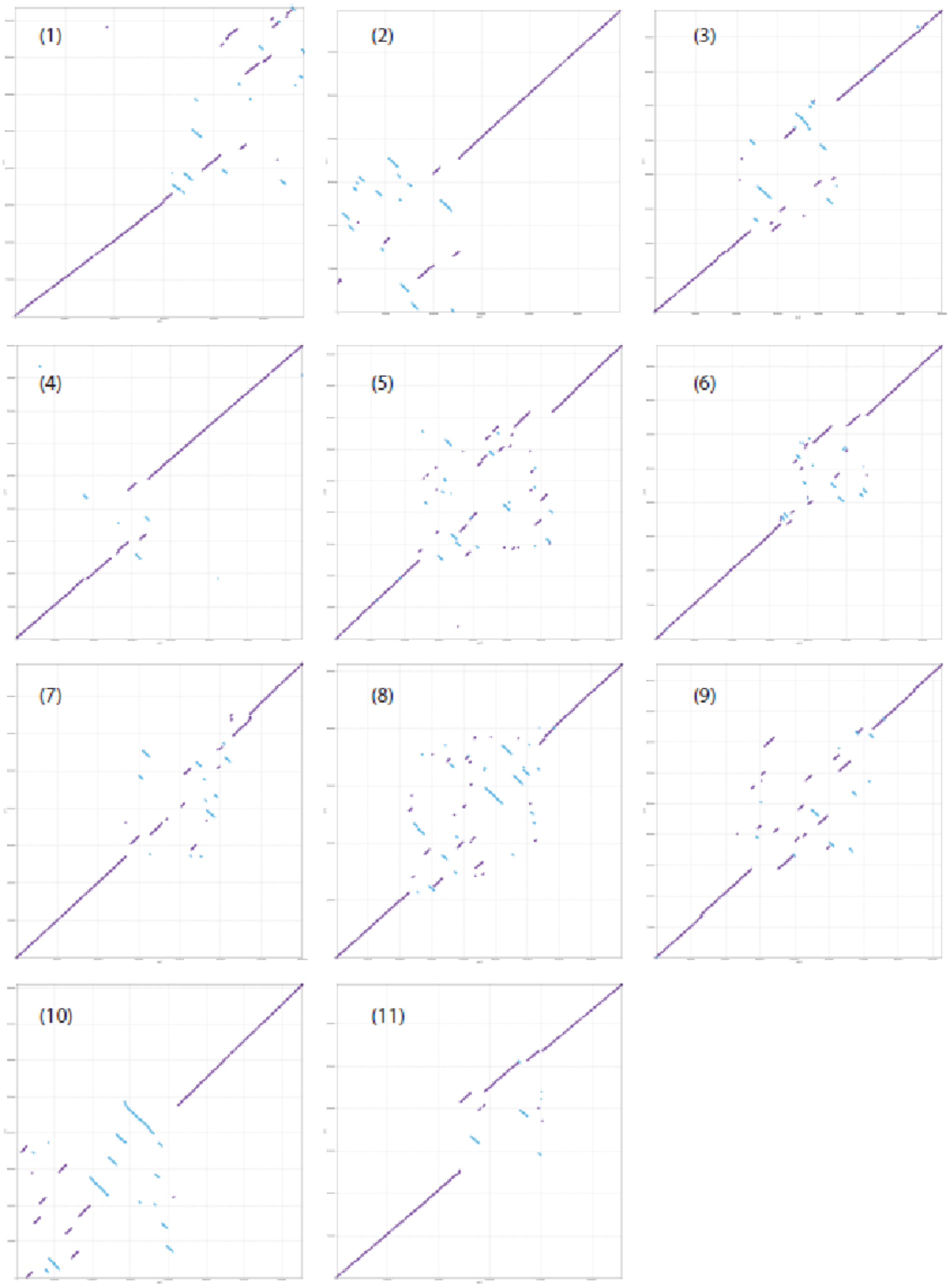

Supplementary Figure 3: Contig flagged as conflictual with the optical maps. The contigs NGS41 (380,641bp) contains in tandem repetitive elements. The contig was split at the position 299,945b in two contigs of 299,945 et 80,696bp.

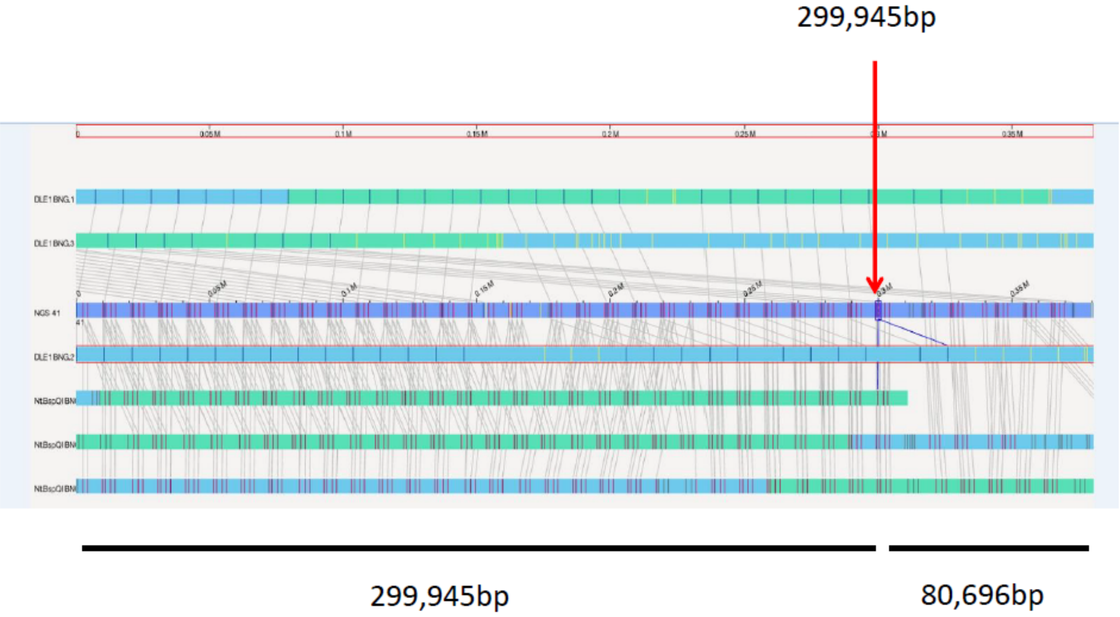

Supplementary Figure 4: Characterization of specific regions of the *Musa acuminata* V4 assembly. Blue bars represent the number of new regions for each chromosome and red crosses represent the maximum size on each chromosome.

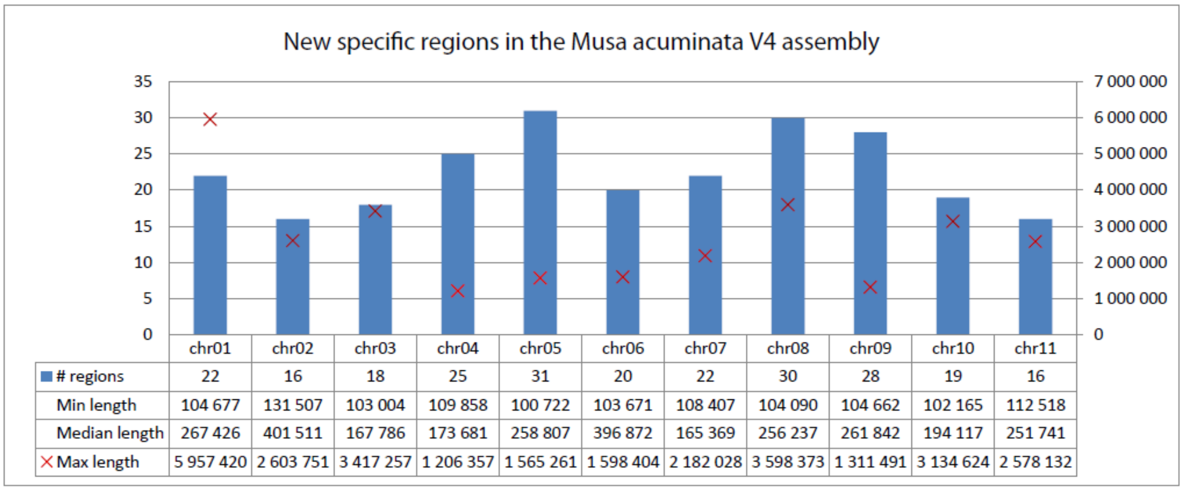

	chr01	chr02	chr03	chr04	chr05	chr06	chr07	chr08	chr09	chr10	chr11
# regions	22	16	18	25	31	20	22	30	28	19	16
Min length	104 677	131 507	103 004	109 858	100 722	103 671	108 407	104 090	104 662	102 165	112 518
Median length	267 426	401 511	167 786	173 681	258 807	396 872	165 369	256 237	261 842	194 117	251 741
Max length	5 957 420	2 603 751	3 417 257	1 206 357	1 565 261	1 598 404	2 182 028	3 598 373	1 311 491	3 134 624	2 578 132

Supplementary Figure 5: Composition of specific regions of the *Musa acuminata* V4 assembly. Proportion of TEs and CDS are in blue and red respectively.

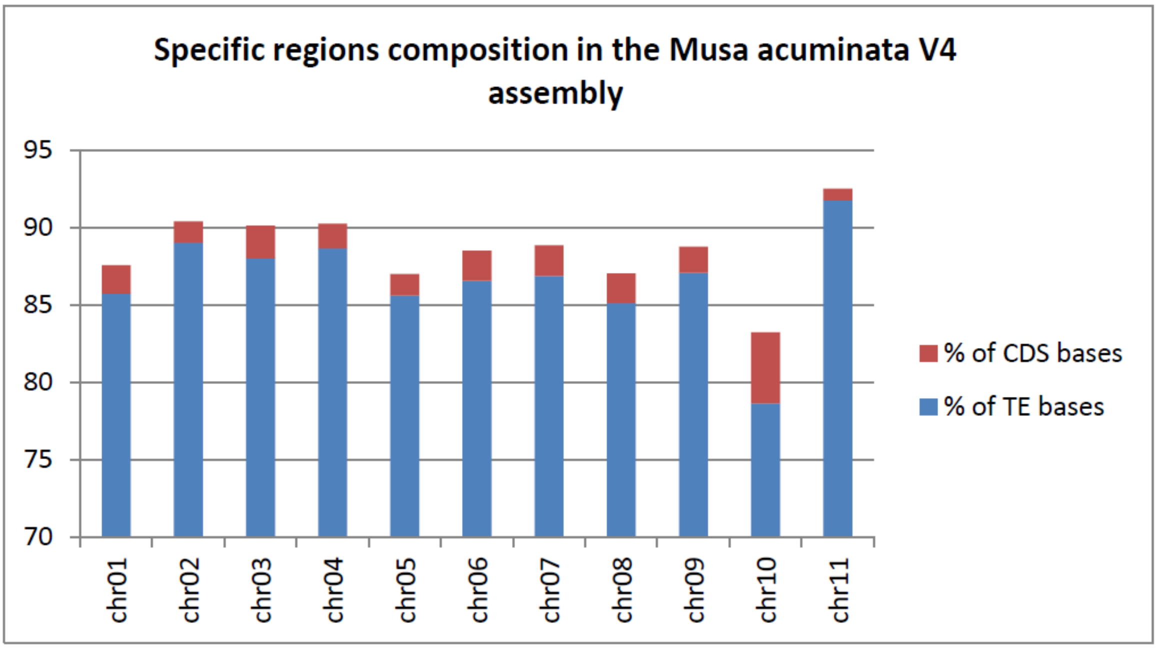

Supplementary Figure 6: Distribution of the repeats along the V4 chromosomes. Proportion of each TE category on each chromosome.

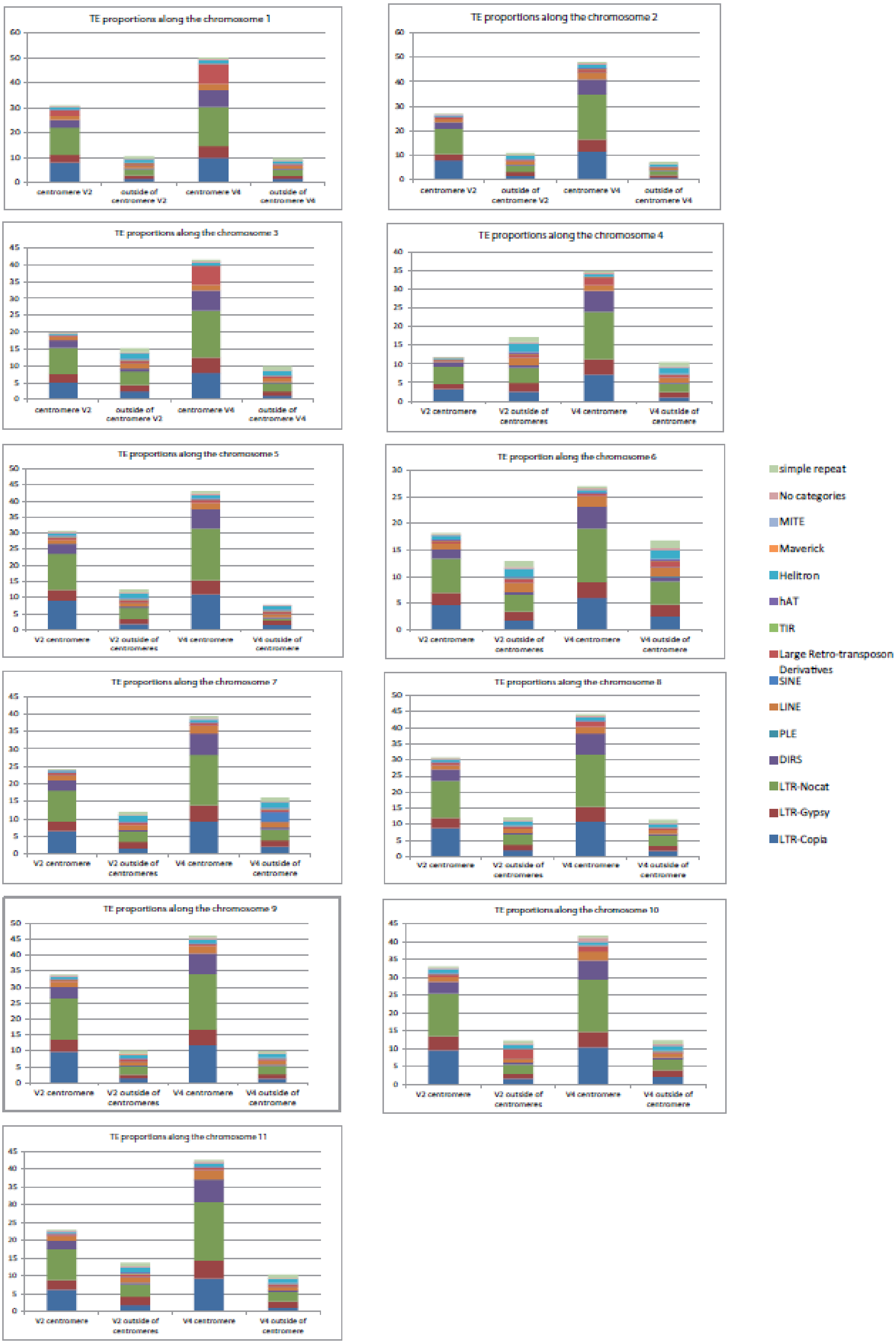

Supplementary Figure 7: Screenshot of the genome browser focusing on a *Musa acuminata* V4 chromosome 1 region. These new annotated genes in the center of track 1 (Gene Predictions) are included in TDGs cluster and were absent from the *Musa acuminata* V2 annotation.

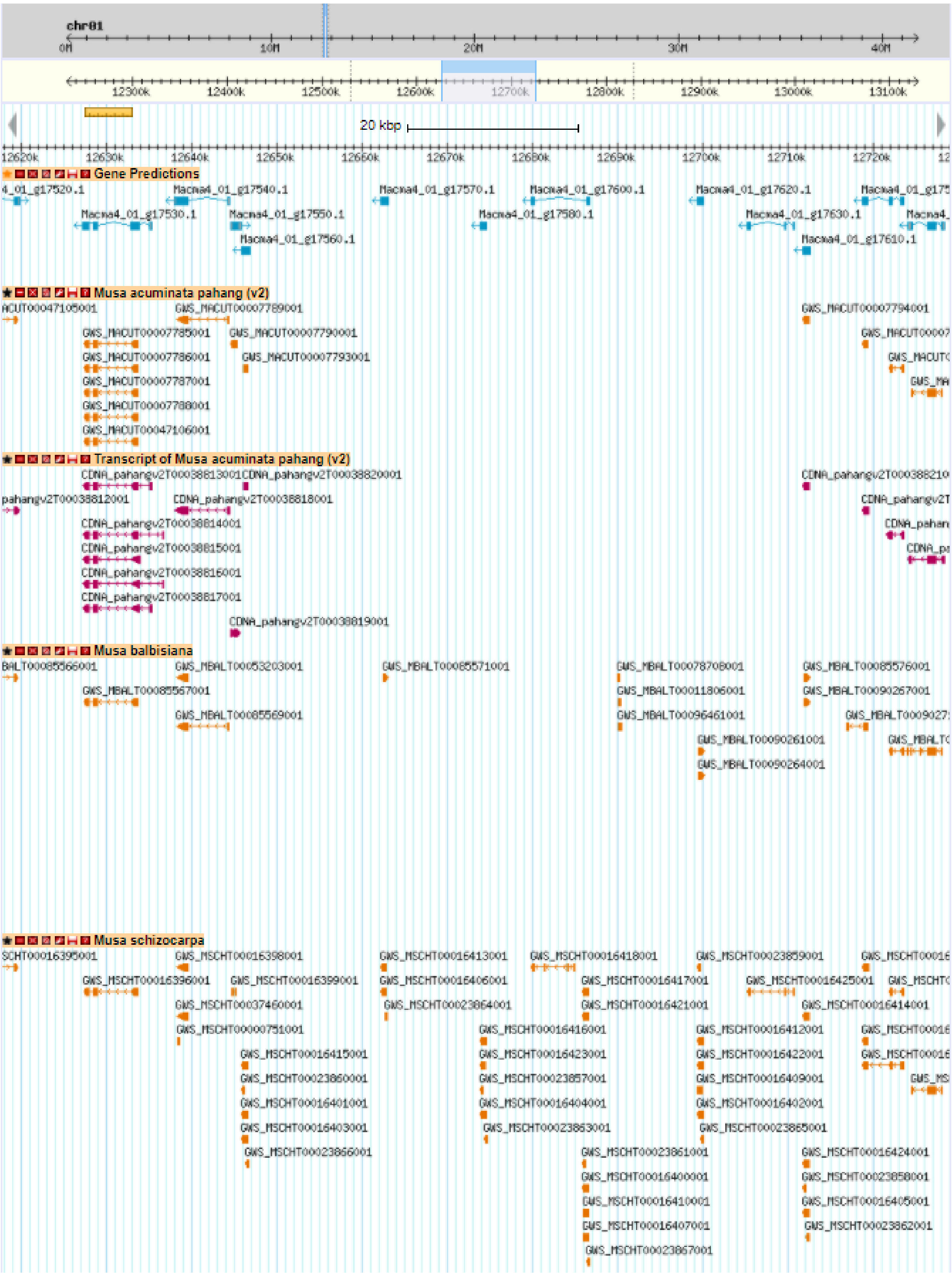

Supplementary Figure 8: Comparison of the structure of NLR loci clusters between DH-Pahang V2 and V4 assemblies. The four panels represent dot plots of NLR loci clusters on chromosomes 3, 7 and 10 as indicated on top of each panel. The predicted NLR loci for each version are represented on the right side and at the bottom of the dot plots by blue boxes. Red boxes represent regions bearing undetermined nucleotides. Region coordinates are also indicated.

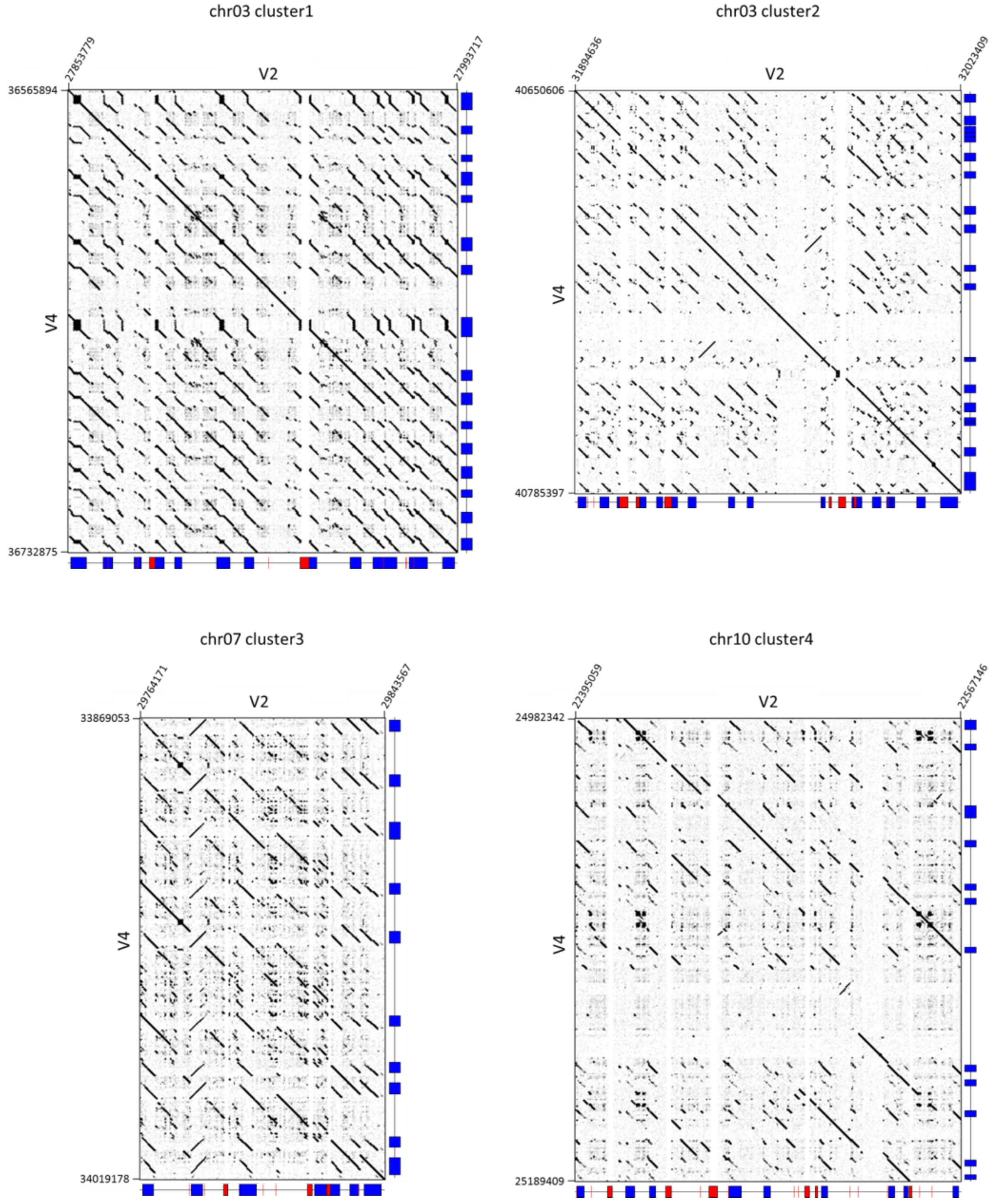

Supplementary Figure 9: Dot plot of *Musa balbisiana* assembly against *Musa acuminata* V4 assembly. *Musa balbisiana* and *acuminata* chromosomes are on the y-axis and x_axis respectively.

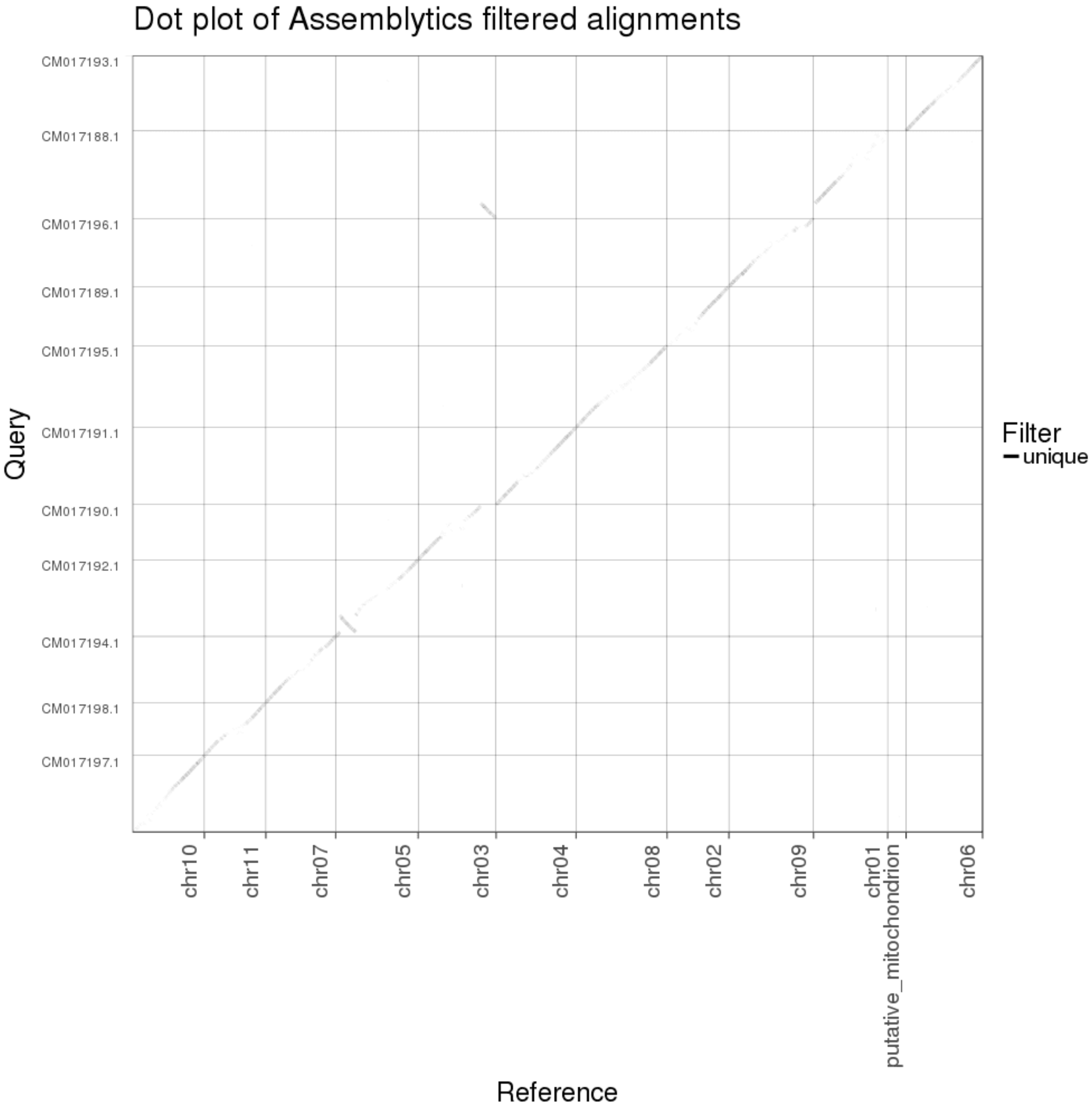

Supplementary Figure 10: Dot plot of *Musa schizocarpa* assembly against *Musa acuminata* V4 assembly. *Musa schizocarpa* and *acuminata* chromosomes are on the y-axis and x_axis respectively.

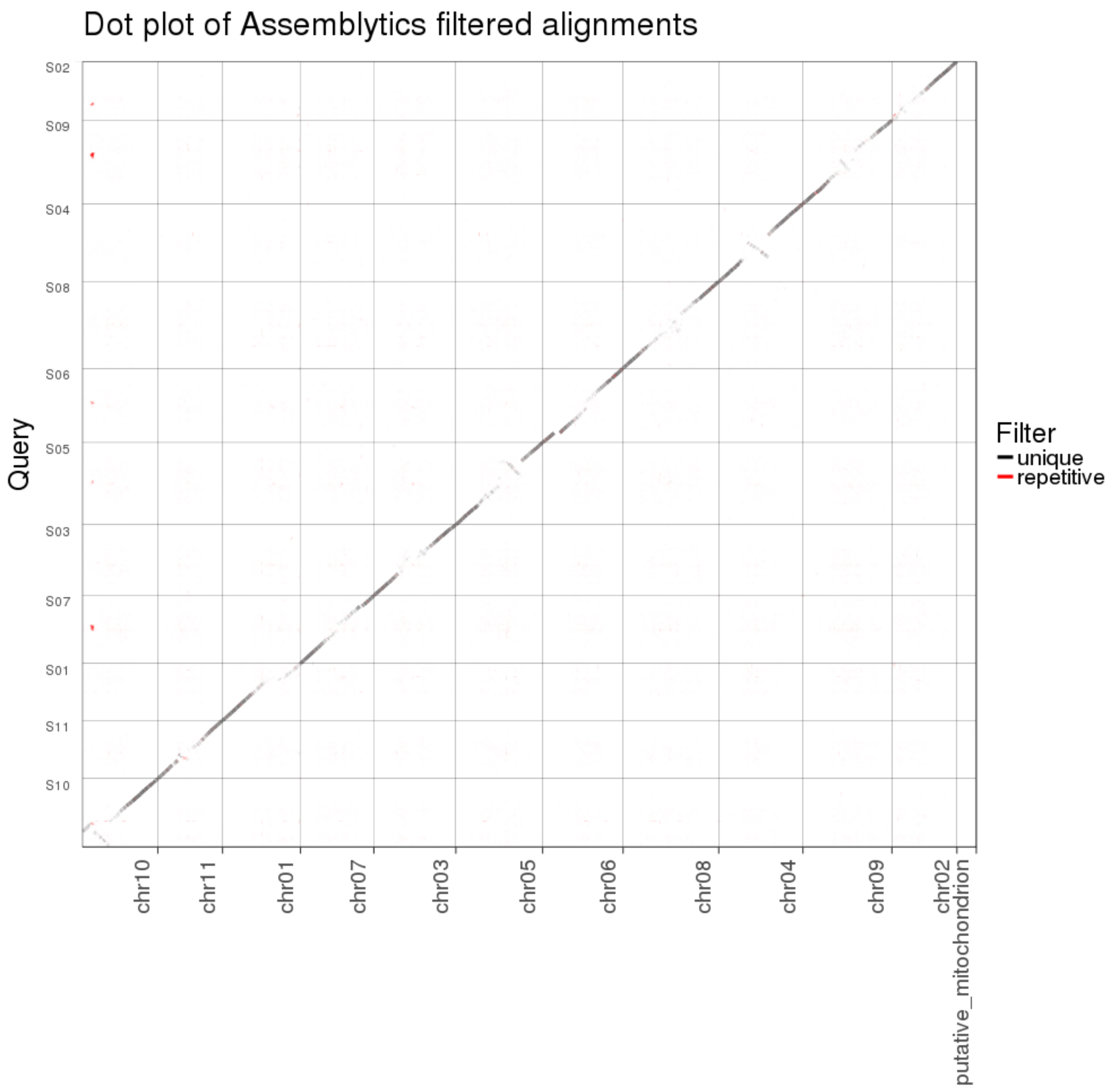

Supplementary Figure 11: Remaining gaps after negative gap resolution. Example of a remaining sized gap in chromosome 1, located between the position 29,171,567 and 29,335,274. The gap was sized thanks to the BspQI map.

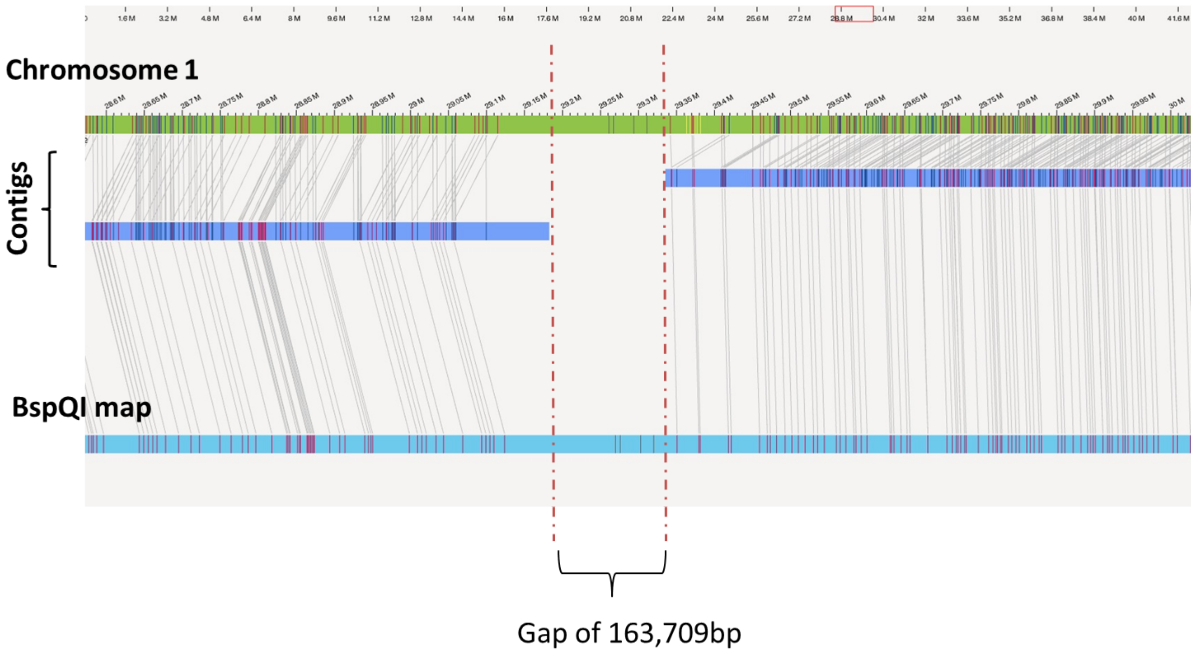

Supplementary Figure 12: gDNA extraction of DH-Pahang. DNA quality was checked on a 2200 TapeStation automated electrophoresis system (Agilent, CA, USA). Ninety seven % of the DNA fragments have a length >50Kb. (a) before removal of small DNA fragments with Short Read Eliminator XL (Circulomics, MD, USA) (b) after removal of small DNA fragments

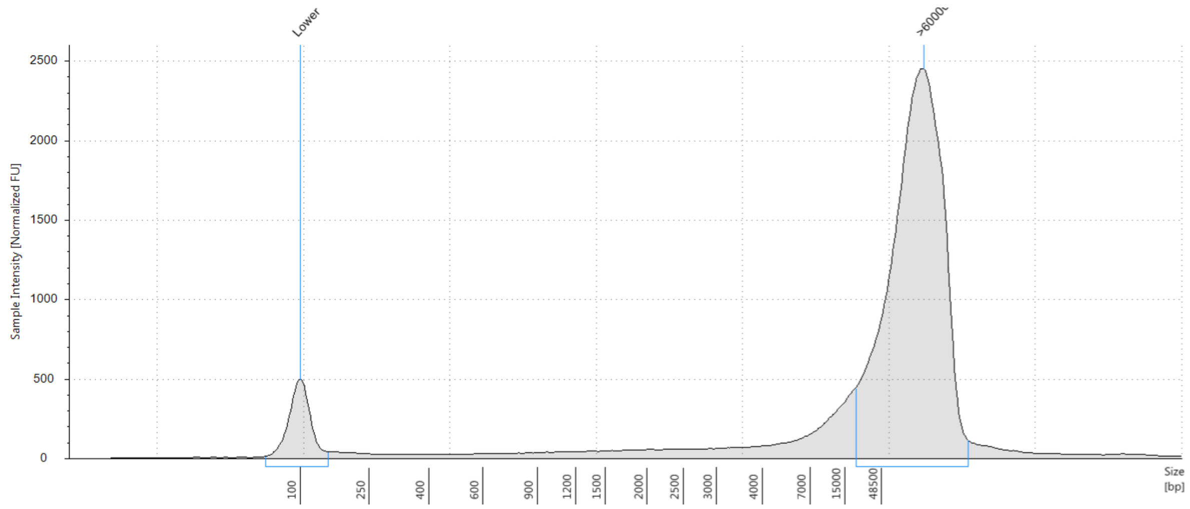

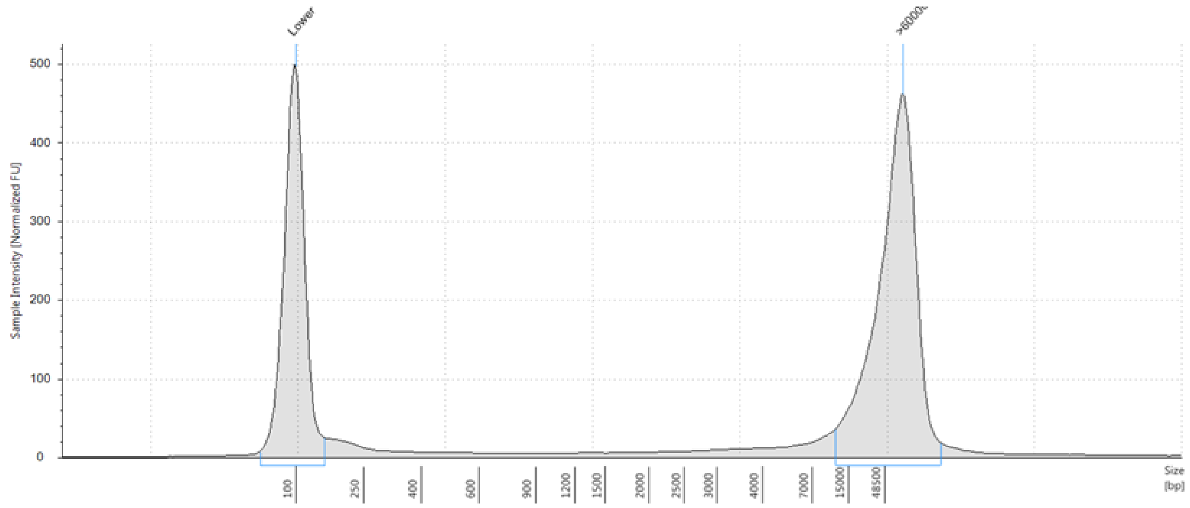

Supplementary Figure 13: Overview of the nanopore contigs in the V4 assembly. Alignment of the two optical maps (a, red) DLE-1 and (b, red) BspQI with the nanopore contigs (c, blue). The vertical grey lines between the contigs and the optical maps represent the matches between enzymatic cuts from the map and the DNA sequences. The number of contigs of each chromosome is indicated.

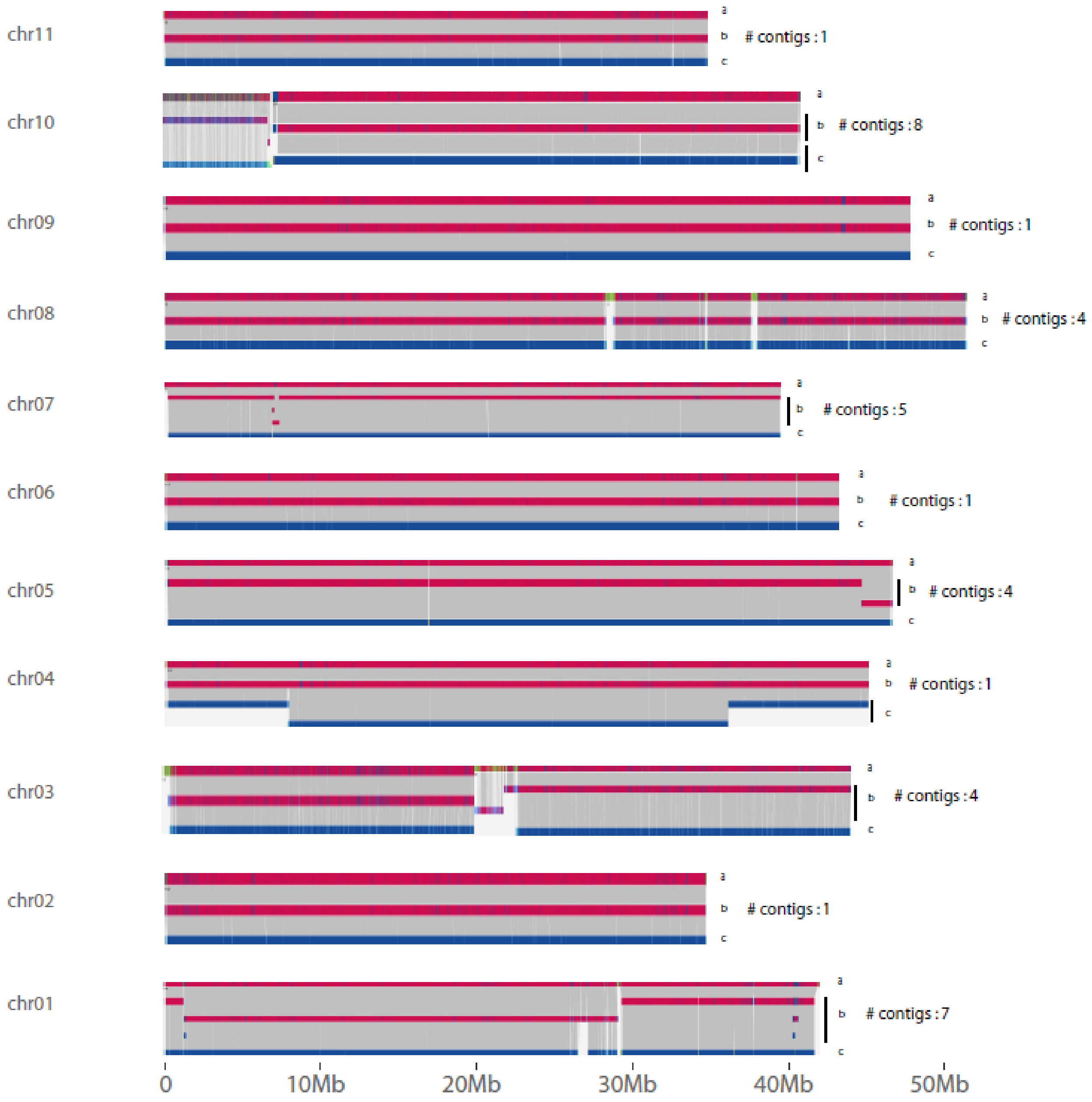

Supplementary Figure 14: Dot plot showing marker linkage along ordered scaffolds of linkage group 01-04. This figure showed marker linkage of linkage group 01-04 which contained markers from chromosome 01 and 04. Because of chromosomal co-segregation due to reciprocal translocation between chromosome 01 and 04, markers from these chromosomes are linked. This resulted by the linkage of markers from a region of scaffold_3 to a region of scaffold_5. Interpretation of the figure suggested that in fact scaffold_3 corresponded to one chromosome and scaffold_5 corresponded to another chromosome.

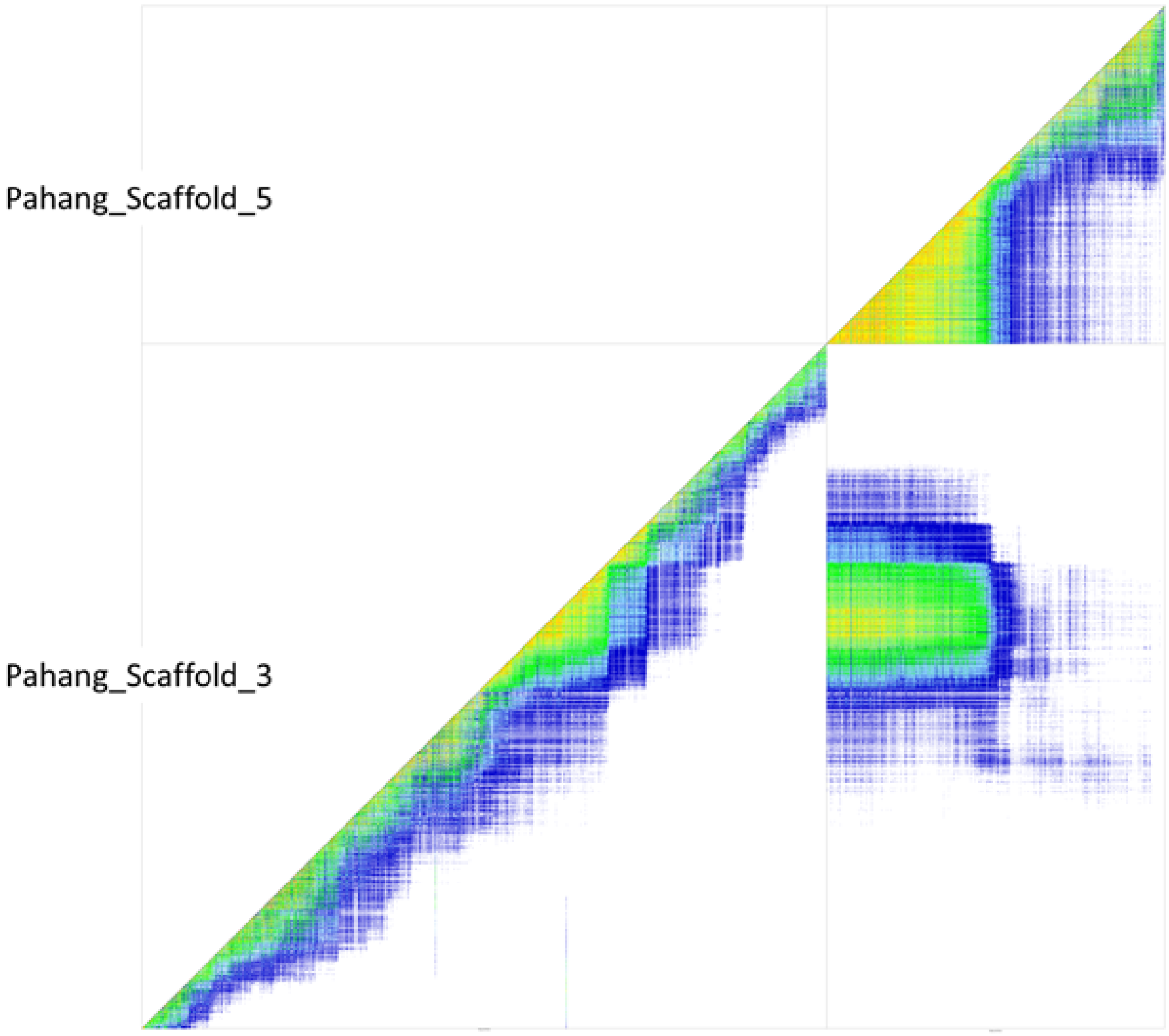

Supplementary Table 1: Contigs details before and after negative gap resolution. Gaps of 100bp are gaps of unknown length generated by the anchoring of contigs using the genetic map. Gaps of 13bp are gaps of unknown size generated by the BioNano pipeline.

DH-Pahang chromosome	# corresponding NECAT contigs	# hybrid scaffolds	# contigs after negative gap resolution	Gaps length (bp)
chr01	7	1	5	163,709 - 13 - 37,440 - 13
chr02	1	1	1	/
chr03	4	2	2	100
chr04	1	1	1	/
chr05	4	1	4	53,161 - 30,868 - 32,283
chr06	1	1	1	/
chr07	5	1	4	13 - 13 - 13
chr08	4	2	4	100 - 46,534 - 324,396
chr09	1	1	1	/
chr10	8	2	2	100
chr11	1	1	1	/

Supplementary Table 2: Assemblies quality scores. Comparison of assembly quality scores of the V1 and V4 assemblies.

Assembly	V1	V4
K-mer completion	95.7068	98.1327
qscore	49.1651	38.7878
qscore on shared region	V1 : 50.1293<br>V4 : 45.9284	

Supplementary Table 3: TE classes proportions in *Musa acuminata* (V2 and V4), *Musa schizocarpa* and *Musa balbisiana*. Transposable elements are classified according to their class.

		Musa acuminata V4 (%)	*Musa acuminata* V2 (%)	*Musa schizocarpa* (%)	*Musa balbisiana* (%)
Class I (retrotransposons)					
LTR	Copia	11,079	8,965	13,766	12,432
	Gypsy	5,986	4,668	5,848	5,137
	no cat	17,788	12,743	19,767	16,547
DIRS	RYX	6,323	3,252	6,094	4,157
PLE	Penelope	0,003	0,003	0,003	0,004
LINE	RIL/RIX	3,492	2,741	3,478	2,941
SINE	RSX	0,005	0,006	0,009	0,004
Large Retro-transposon Derivatives	RXX	2,678	1,347	2,590	1,597
Class II (DNA transposons)- Subclass 1					
TIR	DTX	0,163	0,172	0,180	0,185
hAT	DTA	0,506	0,538	0,525	0,637
Class II (DNA transposons)- Subclass 2					
Helitron	DHH/DHX	2,292	2,175	1,737	2,152
Maverick	DMX	0,005	0,006	0,007	0,006
MITE (miniature inverted repeat transposable elements)	DXX	0,029	0,028	0,023	0,027
No categories		0,722	0,549	1,097	0,762
simple repeat		1,549	1,190	1,221	2,767
	TOTAL	52,623	38,383	56,345	49,353

Supplementary Table 4: Comparison of 5S ribosomal gene clusters in V2 and V4 Musa assemblies. Number of bases in each assembly and on each chromosome tagged as 5S ribosomal genes.

	Base pair covered in V2 assembly (predicted genes)	Base pair covered in V4 assembly (predicted genes)
chr01	0	59,949 (1,882)
chr02	0	0
chr03	194 (3)	179,282 (4,645)
chr04	0	413 (10)
chr05	529 (21)	640 (19)
chr06	68 (1)	68 (1)
chr07	419 (7)	216 (3)
chr08	4,104 (38)	127,057 (1,135)
chr09	547 (14)	80 (1)
chr10	116 (6)	0
chr11	0	0
Un	1,605 (40)	-
Total	7,582 (130)	367,705 (7,696)

Supplementary Table 5: Proportion of new annotated genes in each *Musa acuminata* chromosome of the V4 assembly. Number of annotated genes (first column), duplicated genes (second column), new genes in the V4 assembly (third column) and new genes that are tandemly duplicated (last column).

	Nb of annotated genes	Nb of tandemly duplicated genes	Nb of new annotated genes	Nb of new tandemly duplicated genes
chr01	2,757	397 (14.40%)	369 (13.38%)	173 (46,88%)
chr02	2,680	300 (11.19%)	232 (8.66%)	88 (37,93%)
chr03	3,533	314 (8.89%)	235 (6.65%)	85 (36,17%)
chr04	4,254	343 (8.06%)	258 (6.06%)	66 (25,58%)
chr05	3,345	263 (7.86%)	251 (7.50%)	73 (29,08%)
chr06	4,076	349 (8.56%)	283 (6.94%)	96 (33,92%)
chr07	3,080	324 (10.52%)	190 (6.17%)	83 (43,68%)
chr08	3,604	299 (8.30%)	221 (6.13%)	67 (30,32%)
chr09	3,342	342 (10.23%)	243 (7.27%)	84 (34,57%)
chr10	3,552	527 (14.84%)	510 (14.36%)	250 (49,02%)
chr11	2,612	175 (6.70%)	171 (6.55%)	42 (24,56%)
Total	36,835	3,633 (9.86%)	2,963 (8.04%)	1,137 (38.37%)

Supplementary Table 6: Comparison of NLR clusters between the V2 and V4 assemblies (based on NLR-Annotator predictions). The four NLR clusters are characterized (chromosome, start and end position, size, number of unknown bases, number of genes) in the V2 (four first lines) and V4 assemblies (fout last lines).

Assembly version	Chromosome	Cluster start coordinate	Cluster end coordinate	Size (bp)	Nb. Undetermined nucleotides (N)	Nb. NLR loci detected
V2	chr03	27,854,779	27,992,717	137,938	5,255	14
V2	chr03	31,895,636	32,022,409	126,773	10,472	17
V2	chr07	29,765,171	29,842,567	77,396	4,269	6
V2	chr10	22,396,059	22,566,146	170,087	13,045	9
V4	chr03	36,566,894	36,731,875	164,981	0	16
V4	chr03	40,651,606	40,784,397	132,791	0	18
V4	chr07	33,870,053	34,018,178	148,125	0	10
V4	chr10	24,960,703	25,188,409	227,706	0	13

Supplementary Table 7: Statistics of the ONT datasets. Sequencing metrics of the nanopore long-reads: complete dataset, longest reads only and highest quality reads.

	DH-Pahang		
	Raw reads PromethION	Longest reads	Filtlong reads
Cumulative size	92,749,841,432	13,500,017,777	13,500,035,543
# of reads	5,185,398	175,937	273,413
Coverage	206x	30x	30x
N50 (bp)	31,640	74,681	52,825

Supplementary Table 8: DH-Pahang ONT assembly statistics. Metrics of the assemblies generated using different assemblers and the three nanopore dataset (all reads, longest and highest score).

	SMARTDeNovo			Redbean		
Subset of reads used	All reads	Longest	Filtlong	All reads	Longest	Filtlong
Cumulative size	482,349,453	465,668,591	431,317,546	2,021,646,269	802,627,210	606,906,240
# contigs	121	183	783	43,296	13,785	10,307
N50 (L50)	19,507,988 (9)	5,667,738 (22)	977,146 (111)	75,016 (6,675)	93,433 (1,502)	92,564 (989)
N90 (L90)	2,709,408 (28)	1,176,439 (92)	230,199 (490)	21,284 (26,326)	25,463 (7,959)	25,353 (6,103)

	Flye			NECAT
Subset of reads used	All reads	Longest	Filtlong	All reads
Cumulative size	470,287,666	471,731,899	439,685,078	485,698,222
# contigs	449	318	953	175
N50 (L50)	15,404,016 (12)	12,916,122 (13)	1,521,384 (73)	32,031,704 (7)
N90 (L90)	2,621,850 (40)	3,315,873 (37)	298,601 (333)	5,650,309 (17)

Supplementary Table 9: DH-Pahang bionano dataset. Statistics of the molecule used to generate the two optical maps.

	DLE-1 Molecule statistics	BspQI Molecule statistics
Total number of molecules	2,151,406	2,897,832
Total length (Mbp)	209,178.556	398,318,111
Average length (kbp)	97.229	137.109
Molecule N50 (kbp)	129.750	220.875
Label density (/100kb)	13.709	10.502
Number of Flow cell	2	1

Supplementary Table 10: DH-Pahang bionano genome map. Statistics of the two optical maps obtained with the DLE and BspQI enzymes.

	DLE-1 genome map	BspQI genome map
Genome map number	24	71
Total Genome Map Length (Mbp)	469.764	474.019
Genome Map N50 (Mbp)	35.022	16.002

Supplementary Table 11: DH-Pahang hybrid scaffolding and polishing. Statistics of the raw nanopore assembly (first column), the assembly obtained by combining nanopore contigs and optical maps (second, third and fourth column), and the final assembly after the resolution of negative gaps (last column).

	nanopore contigs polished	Hybrid scaffolds	contigs not scaffolded	final hybrid scaffolds (hybrid scaffolds + contigs not scaffolded)	scaffolds after negative gap resolution and polishing
number	124	16	80	96	97
N50 (L50)	32,091,274 (7)	39,508,388 (6)	249,463 (18)	39,508,388 (6)	39,373,400 (6)
N90 (L90)	5,668,018 (17)	21,536,064 (12)	87,718 (59)	21,536,064 (12)	21,536,112 (12)
maxSize	47,719,325	47,719,325	673,878	47,719,325	47,719,527
Assembly size	485,318,484	471,709,278	14,435,609	486,144,887	484,747,212
% of N	0%	0.18%	0%	0.17%	0.14%

Supplementary Table 12: **Marker number per scaffold and linkage group.** Number of markers aligned on each scaffolds (lines) for each linkage groups (columns).

scaffold	LG0 1-04	LG0 2	LG0 3	LG0 5	LG0 6	LG0 7	LG0 8	LG0 9	LG1 0	LG1 1
Pahang_Scaffold_1	0	0	0	1	0	1	0	2089	0	0
Pahang_Scaffold_2	0	0	0	1874	0	1	0	0	1	0
Pahang_Scaffold_3	2191	1	0	0	0	0	0	1	0	0
Pahang_Scaffold_4	0	0	1	0	2190	0	0	1	0	0
Pahang_Scaffold_5	1082	0	2	3	0	0	2	1	0	0
Pahang_Scaffold_6	0	0	0	1	0	1687	0	0	1	1
Pahang_Scaffold_7	0	0	0	0	1	0	0	0	1784	0
Pahang_Scaffold_8	0	1483	0	0	0	0	0	0	0	0
Pahang_Scaffold_9	0	0	0	1	0	0	1	1	0	1500
Pahang_Scaffold_10	0	0	0	1	0	0	1283	0	0	0
Pahang_Scaffold_11	0	0	1027	1	0	0	0	0	0	0
Pahang_Scaffold_12	0	0	0	0	0	0	1276	0	0	0
Pahang_Scaffold_13	0	0	850	0	0	0	1	0	1	0
Pahang_Scaffold_14	0	0	0	1	0	0	0	0	173	0
Pahang_Scaffold_59	1	0	0	0	0	0	0	0	0	0
