# Supplementary material for: Telomere-to-telomere gapless chromosomes of banana using nanopore sequencing
Source: Commun Biol. 2021 Sep 7;4:1047. doi: 10.1038/s42003-021-02559-3 (PMC8423783; doi:10.1038/s42003-021-02559-3)
Supplement: Supplementary file 3 — Description of Supplementary Files [file 42003_2021_2559_MOESM3_ESM.pdf]

Description of Additional Supplementary Files

File name: Supplementary Data 1

Description: Annotated NLR genes in the V4 assembly of Musa acuminata (DH-Pahang).

File name: Supplementary Data 2

Description: Annotated NLR genes in the V2 assembly of Musa acuminata (DH-Pahang).
